# Circulating Haptoglobin and Metabolic Syndrome in Renal Transplant Recipients

**DOI:** 10.1038/s41598-017-14302-2

**Published:** 2017-10-27

**Authors:** Isidor Minović, Michele F. Eisenga, Ineke J. Riphagen, Else van den Berg, Jenny Kootstra-Ros, Anne-Roos S. Frenay, Harry van Goor, Gerald Rimbach, Tuba Esatbeyoglu, Andy P. Levy, Carlo A. J. M. Gaillard, Johanna M. Geleijnse, Manfred L. Eggersdorfer, Gerjan J. Navis, Ido P. Kema, Stephan J. L. Bakker

**Affiliations:** 1 Department of Internal Medicine, Division of Nephrology, University of Groningen, University Medical Center Groningen, Hanzeplein 1, 9700 RB Groningen, The Netherlands; 2 Top Institute Food and Nutrition, Nieuwe Kanaal 9-A, 6709 PA Wageningen, The Netherlands; 3 Department of Laboratory Medicine, University of Groningen, University Medical Center Groningen, Hanzeplein 1, 9700 RB Groningen, The Netherlands; 4 Department of Pathology, University of Groningen, University Medical Center Groningen, Hanzeplein 1, 9700 RB Groningen, The Netherlands; 5 Institute of Human Nutrition and Food Science, Christian-Albrechts-University of Kiel, Kiel, Germany; 6 Faculty of Medicine, Technion Institute of Technology, Efron Street 1, Haifa, Israel; 7 Division of Human Nutrition, Wageningen University, Droevendaalsesteeg 4, 6708 PB Wageningen, The Netherlands; 8 DSM Nutritional Products, Wurmisweg 576, 4303 Kaiseraugst, Switzerland

## Abstract

Haptoglobin (Hp) is an acute phase protein that has recently been linked to components of the metabolic syndrome (MetS). We aimed to evaluate Hp as marker of MetS, and to assess its association with long-term outcome in renal transplant recipients (RTR). We measured plasma Hp in a prospective cohort of 699 stable RTR and 149 healthy controls. Median plasma Hp concentration in RTR was 1.4 [interquartile range (IQR), 1.0–1.8] g/L, which was higher compared to 1.1 [0.9–1.4] g/L in controls (P < 0.001). Hp was independently associated with the MetS (β = 0.10) (P = 0.005). During follow-up of 5.4 [4.8–6.1] years, 150 (21%) recipients died, of whom 60 (9%) due to cardiovascular causes, and 83 (12%) RTR developed graft failure. High (≥2.0 g/L) and low (≤0.9 g/L) plasma Hp were associated with increased risk of mortality (HR’s 2.3 [1.3–4.1] and 1.9 [1.0–3.5], resp.), predominantly cardiovascular. The association of high Hp lost significance upon adjustment for inflammation markers (HR 1.5 [0.8–2.7]), while low Hp was independently associated with mortality (HR 2.2 [1.2–4.0]). Hp was not associated with graft failure (P = 0.49). In conclusion, plasma Hp is independently associated with MetS in RTR. Importantly, high and low Hp are associated with increased mortality risk, independent of MetS.

## Introduction

Haptoglobin (Hp) is a hepatic glycoprotein that is traditionally used to monitor *in vivo* hemolysis^1^. As such, Hp is assessed in transplant recipients as marker of calcineurin-induced microangiopathy short after transplantation. However, the role of Hp in transplant recipients under non-hemolytic circumstances, e.g. late after transplantation, has not been fully evaluated.

Previous studies describing the non-hemolytic significance of Hp have primarily been cross-sectional in nature and have mainly focused on the role of Hp in inflammation^2–4^. These studies have established Hp as a positive acute phase protein as a result of induced gene transcription by pro-inflammatory cytokines.

Besides inflammation, Hp has recently gained additional attention as it has been related to key components of the metabolic syndrome (MetS)^5–11^. The MetS is increasingly prevalent among renal transplant recipients (RTR) where it has been found to be an independent risk factor for development of post-transplantation diabetes mellitus, cardiovascular disease, mortality, and graft failure^12,13^. Since Hp has been related to key components of the MetS, Hp could possibly be interpreted as a marker of the MetS. However, as the MetS per se is associated with inflammation^14,15^, it is difficult to distinguish to what extent the previously found associations of Hp with components of the MetS are attributable to inflammation. Furthermore, it is not known whether Hp is associated with long-term outcome in RTR and it is also not known whether potential prospective associations of Hp are influenced by the MetS or inflammation.

In this study, we therefore aimed to cross-sectionally evaluate Hp as marker of the MetS, and to prospectively assess the association of Hp with long-term outcome in RTR, while taking into account the MetS and inflammation.

## Results

### Baseline Characteristics

Median Hp concentration in RTR (age 53 ± 13 years, 57% male) was 1.4 [1.0–1.8], which was significantly higher compared to 1.1 [0.9–1.4] g/L in healthy controls (age 52 ± 10 years, 52% male) (P < 0.001). In multivariable linear regression models, this difference in Hp remained significant after adjustment for age, sex, smoking, Hp genotype, estimated glomerular filtration rate (eGFR), and high-sensitivity C-reactive protein (hs-CRP) (P = 0.002). A list of the available baseline characteristics of healthy controls can be found in Supplementary Table 1.

Median time between transplantation and inclusion of RTR in the study was 5.4 [1.9–12.0] years. Baseline characteristics for the total RTR cohort and across Hp quintiles are shown in Table 1. In RTR at baseline, Hp was positively associated with age, weight, body mass index (BMI), waist circumference, proteinuria, thrombocytes, heart rate, use of antihypertensive drug, hs-CRP, procalcitonin, low density lipoprotein (LDL) cholesterol, triglycerides, use of statin, Hp 1–2 genotype, serum glucose, HbA1c, diabetes, use of antidiabetic drug, alkaline phosphatase, gamma glutamyl transferase, and dose of prednisolone (all P ≤ 0.05). Inverse associations were observed between Hp and eGFR, mean corpuscular volume (MCV), total hemoglobin (Hb), vitamin B6, serum albumin, high density lipoprotein (HDL)-cholesterol, Hp 2–2 genotype, serum iron, transferrin saturation (TSAT), and aspartate aminotransferase (ASAT) (all P ≤ 0.01). The MetS was present in 456 (65%) of 699 RTR and was positively associated with circulating Hp (P < 0.001).

**Table 1:** Baseline characteristics of 699 renal transplant recipients, stratified by haptoglobin quintiles. Hp, haptoglobin; BMI, body mass index; Rtx, renal transplantation; eGFR, estimated glomerular filtration rate; MCV, mean corpuscular volume; Hb, hemoglobin; SBP, systolic blood pressure; DBP, diastolic blood pressure; hs-CRP, high-sensitivity C-reactive protein; HDL, high density lipoprotein; LDL, low density lipoprotein; MetS, metabolic syndrome; Hp, haptoglobin; TSAT, transferrin saturation, ALAT, alanine aminotransferase; ASAT, aspartate aminotransferase; MDA, malondialdehyde; R-SH, serum free thiols.

	Total cohort (n = 699)	Quintiles of Hp					
		Q_1_ ≤ 0.9 g/L (n = 133)	Q_2_ 1.0–1.2 g/L (n = 148)	Q_3_ 1.3–1.5 g/L (n = 123)	Q_4_ 1.6–1.9 g/L (n = 161)	Q_5_ ≥ 2.0 g/L (n = 127)	P_trend_
**Demographics**							
Age, years	53 ± 13	50 ± 13	51 ± 13	54 ± 13	54 ± 12	56 ± 13	<0.001
Male gender, n (%)	398 (57)	74 (55)	80 (54)	76 (61)	95 (58)	73 (57)	0.22
Weight, kg	80 ± 17	75 ± 14	81 ± 16	80 ± 18	83 ± 16	83 ± 16	<0.001
BMI, kg/m^2^	26.0 [23.2–29.3]	24.7 [22.4–26.8]	26.0 [23.3–29.4]	25.8 [23.1–28.9]	26.6 [23.3–30.5]	27.3 [24.2–30.6]	<0.001
BMI<21 kg/m^2^, n (%)	58 (8)	18 (13)	16 (11)	5 (4)	12 (7)	7 (6)	0.01
Waist circumference, cm	101 ± 11	97 ± 9	101 ± 11	100 ± 10	102 ± 10	105 ± 12	<0.001
Smoking status, n (%)							
Current	91 (13)	11 (8)	19 (13)	12 (10)	26 (16)	18 (14)	
Past	322 (46)	62 (46)	63 (42)	65 (52)	60 (37)	69 (54)	0.26
Never	294 (42)	62 (46)	67 (45)	46 (37)	77 (47)	41 (32)	0.07
Time since Rtx, years	5.4 [1.9–12.0]	5.8 [3.3–9.3]	5.3 [1.3–10.9]	5.1 [1.5–10.7]	5.1 [1.5–12.0]	6.1 [2.8–13.9]	0.60
eGFR, mL/min/1,73 m^2^	45 ± 19	48 ± 19	46 ± 17	46 ± 18	43 ± 20	42 ± 19	0.002
Proteinuria, n (%)	161 (23)	23 (17)	28 (19)	24 (19)	44 (27)	40 (31)	0.001
Rejection, n (%)	189 (27)	32 (24)	39 (26)	30 (24)	42 (26)	42 (33)	0.14
Thrombocytes, 10E9/L	237 ± 69	218 ± 59	228 ± 64	235 ± 65	244 ± 68	259 ± 83	<0.001
MCV, fL	90.4 ± 5.9	91.1 ± 5.9	90.4 ± 5.9	90.7 ± 5.9	90.8 ± 6.1	89.3 ± 5.6	0.001
Total Hb, mmol/L	8.2 ± 1.1	8.6 ± 1.2	8.2 ± 1.0	8.3 ± 1.0	8.1 ± 1.1	8.0 ± 1.0	0.007
Parathyroid hormone, pmol/L	8.9 [5.9–14.7]	8.4 [5.6–13.4]	8.9 [5.2–12.9]	8.9 [6.5–15.6]	10.5 [5.8–17.3]	8.9 [6.1–16.3]	0.14
Venous pH	7.37 ± 0.04	7.37 ± 0.04	7.40 ± 0.04	7.37 ± 0.04	7.36 ± 0.04	7.37 ± 0.04	0.53
Venous HCO_3_^−^, mmol/L	24.6 ± 3.1	24.5 ± 3.1	24.8 ± 3.1	25.1 ± 3.0	24.5 ± 3.2	24.2 ± 2.9	0.23
**Dietary intake and nutritional status**							
Total protein intake, g/d	82 ± 20	82 ± 21	83 ± 20	81 ± 20	83 ± 16	83 ± 16	0.77
Animal protein intake, g/d	51 ± 15	50 ± 15	52 ± 16	52 ± 14	52 ± 15	52 ± 15	0.37
Plant protein intake, g/d	31 ± 10	32 ± 11	31 ± 10	30 ± 10	30 ± 9	29 ± 9	0.05
Energy intake, kCal/d	2172 ± 640	2220 ± 679	2193 ± 613	2142 ± 605	2167 ± 576	2093 ± 627	0.17
Vitamin B12, pmol/L	288 [222–377]	304 [225–385]	301 [223–401]	284 [228–357]	271 [210–350]	292 [218–396]	0.29
Vitamin B6, nmol/L	29 [17–50]	31 [17–50]	33 [21–57]	30 [18–48]	27 [16–47]	23 [13–47]	0.001
Folic acid, nmol/L	18 [14–26]	18 [14–26]	19 [14–27]	19 [13–25]	18 [14–26]	18 [14–28]	0.67
**Cardiovascular parameters**							
SBP, mmHg	136 ± 18	133 ± 17	137 ± 16	138 ± 19	135 ± 18	137 ± 17	0.29
DBP, mmHg	83 ± 11	81 ± 11	85 ± 11	83 ± 11	82 ± 12	82 ± 9	0.58
Heart rate, bpm	69 ± 12	66 ± 10	68 ± 12	69 ± 14	69 ± 11	72 ± 12	<0.001
Use of antihypertensive drug, n (%)	615 (88)	108 (80)	131 (88)	110 (89)	148 (91)	120 (94)	<0.001
Hs–CRP, mg/L	1.6 [0.7–4.6]	0.8 [0.4–1.8]	1.3 [0.6–2.5]	1.5 [0.7–3.3]	1.8 [0.8–4.8]	6.0 [2.1–13.6]	<0.001
Procalcitonin, µ/L	0.05 [0.03–0.08]	0.05 [0.02–0.08]	0.04 [0.02–0.06]	0.04 [0.03–0.07]	0.05 [0.03–0.07]	0.06 [0.03–1.0]	<<0.001
Serum albumin, g/L	43.0 ± 2.9	43.4 ± 3.2	43.4 ± 2.6	43.5 ± 2.9	42.7 ± 2.9	41.6 ± 3.3	<0.001
Total cholesterol, mmol/L	5.1 ± 1.1	4.9 ± 1.0	5.1 ± 1.1	5.1 ± 1.1	5.3 ± 1.1	5.2 ± 1.3	0.05
HDL-cholesterol, mmol/L	1.4 ± 0.5	1.5 ± 0.5	1.4 ± 0.5	1.4 ± 0.5	1.4 ± 0.5	1.3 ± 0.4	<0.001
LDL-cholesterol, mmol/L	2.9 [2.3–3.5]	2.7 [2.2–3.3]	3.0 [2.4–3.4]	2.9 [2.5–3.5]	2.9 [2.3–3.7]	2.8 [2.2–3.7]	0.03
Triglycerides, mmol/L	1.7 [1.3–2.3]	1.4 [1.1–1.9]	1.6 [1.2–2.3]	1.7 [1.2–2.2]	1.7 [1.3–2.5]	2.1 [1.5–2.9]	<0.001
Use of statin, n (%)	370 (53)	69 (51)	67 (45)	64 (52)	85 (52)	84 (66)	0.01
MetS, n (%)	456 (65)	70 (52)	95 (64)	79 (64)	113 (69)	99 (77)	<0.001
Hemolytic index > 20, n (%)	7 (1)	1 (1)	1 (1)	1 (1)	2 (1)	1 (1)	0.73
Hp 1-1 genotype	126 (18)	9 (7)	12 (8)	31 (25)	33 (20)	36 (28)	
Hp 1-2 genotype	350 (50)	55 (41)	75 (50)	69 (56)	86 (53)	63 (49)	0.004
Hp 2-2 genotype	231 (33)	70 (52)	61 (41)	24 (19)	44 (27)	29 (23)	<0.001
**Glucose homeostasis parameters**							
Serum glucose, mmol/L	5.3 [4.8–6.0]	5.1 [4.7–5.7]	5.2 [4.8–6.0]	5.2 [4.7–5.8]	5.3 [4.8–6.2]	5.4 [4.8–7.0]	0.001
HbA_1c_, n (%)	6.0 ± 0.8	5.8 ± 0.8	5.9 ± 0.6	5.9 ± 0.7	6.1 ± 0.8	6.5 ± 1.1	<0.001
Diabetes, n (%)	168 (24)	22 (16)	27 (18)	24 (19)	22 (26)	55 (43)	<0.001
Use of antidiabetic drug, n (%)	112 (16)	16 (12)	15 (10)	17 (14)	24 (15)	38 (30)	<0.001
**Iron metabolism parameters**							
Serum iron, µmol/L	15.3 ± 6.0	16.9 ± 7.1	15.6 ± 5.6	15.9 ± 6.4	14.9 ± 5.1	12.9 ± 5.1	<0.001
Transferrin, g/L	2.5 ± 0.3	2.5 ± 0.3	2.6 ± 0.5	2.5 ± 0.4	2.5 ± 0.4	2.4 ± 0.4	0.19
TSAT, %	25.1 ± 11.7	27.9 ± 11.9	25.2 ± 10.4	26.2 ± 11.0	24.8 ± 10.2	21.6 ± 8.8	<0.001
**Liver function parameters**							
ALAT, U/L	19 [14–26]	19 [14–28]	19 [15–27]	19 [15–24]	19 [15–24]	19 [14–27]	0.60
ASAT, U/L	22 [18–27]	24 [19–30]	23 [19–27]	22 [18–27]	21 [18–26]	20 [16–26]	<0.001
Alkaline phosphatase, U/L	67 [53–83]	63 [48–81]	65 [53–79]	66 [54–81]	69 [57–87]	70 [56–93]	0.001
Gamma glutamyl transferase, U/L	26 [19–41]	26 [17–41]	25 [17–46]	25 [19–35]	28 [20–39]	30 [19–46]	0.03
**Antioxidant status parameters**							
Plasma MDA, µmol/L	2.6 [1.9–3.8]	2.8 [2.0–4.1]	2.6 [2.1–3.7]	2.3 [1.8–3.1]	2.4 [1.9–4.4]	2.4 [1.8–3.7]	0.41
Urinary MDA excretion, µmol/24 h	9.7 [5.9–15.7]	9.6 [6.0–14.2]	9.3 [5.6–15.9]	9.6 [6.2–16.0]	10.3 [6.0–15.4]	9.4 [5.5–16.1]	0.12
R-SH/total protein, mM/g	1.83 ± 0.67	1.80 ± 0.64	1.91 ± 0.74	1.81 ± 0.59	1.75 ± 0.72	1.90 ± 0.60	0.80
**Immunosuppression**							
Use of calcineurin inhibitor, n (%)							
-Cyclosporine	273 (39)	46 (34)	61 (41)	50 (40)	70 (43)	49 (38)	0.33
-Tacrolimus	133 (19)	26 (19)	36 (24)	19 (15)	26 (16)	19 (15)	0.59
Use of proliferation inhibitor, n (%)	580 (83)	116 (86)	125 (84)	104 (84)	135 (83)	102 (80)	0.16
Prednisolone dose < 10 mg/24 h, n (%)	288 (41)	64 (47)	73 (49)	49 (40)	60 (37)	42 (33)	0.001

Data from univariable and multivariable linear regression models demonstrating the associations of Hp with components of the MetS, are presented in Table 2. Adjustment for age, sex, smoking status, Hp genotype, eGFR, proteinuria, and use of prednisolone lowered the standardized beta of antihypertensive drug use by 38%, but did not materially affect the associations of Hp with other components of the MetS. However, additional adjustment for hs-CRP, procalcitonin, and serum albumin lowered the standardized betas of the association of Hp with waist circumference, triglycerides, serum glucose, antidiabetic drug use, and the MetS overall by 45%, 31%, 42%, 21%, and 29%, respectively. Conversely, adjustment for the inflammation parameters increased the standardized beta of HDL-cholesterol and statin use by 54% and 75%, respectively. Importantly, in this multivariable model Hp remained associated with components of the MetS (P < 0.05), as with the MetS overall (P = 0.005).

**Table 2:** Univariable and multivariable associations of MetS components with Hp. *Crude associations; **adjusted for age, sex, smoking, Hp genotype, eGFR, proteinuria, and use of prednisolone; ***as model 2, additionally adjusted for hs-CRP, procalcitonin, and serum albumin. Stand. beta, standardized beta; SBP, systolic blood pressure; DBP, diastolic bloodpressure; HDL, high-density lipoprotein; MetS, metabolic syndrome.

	Model 1*		Model 2**		Model 3***	
	Stand. beta	P-value	Stand. beta	P-value	Stand. beta	P-value
Waist circumference, cm	**0.22**	<**0.001**	**0.22**	<**0.001**	**0.12**	**0.001**
SBP, mmHg	0.03	0.49	−0.03	0.41	0.01	0.94
DBP, mmHg	0.01	0.81	−0.02	0.71	−0.01	0.86
Antihypertensive drugs, n (%)	**0.16**	<**0.001**	**0.10**	**0.01**	**0.08**	**0.02**
HDL-cholesterol, mmol/L	−**0.12**	**0.002**	−**0.13**	**0.001**	−0.06	0.14
Triglycerides, mmol/L	**0.19**	<**0.001**	**0.16**	<**0.001**	**0.11**	**0.002**
Statin, n (%)	**0.09**	**0.02**	**0.08**	**0.04**	**0.14**	<**0.001**
Serum glucose, mmol/L	**0.11**	**0.005**	**0.12**	**0.002**	**0.07**	**0.05**
Antidiabetic drugs, n (%)	**0.15**	<**0.001**	**0.14**	<**0.001**	**0.11**	**0.002**
*MetS, n (%)*	***0.16***	<***0.001***	***0.14***	<***0.001***	***0.10***	***0.005***

### Hp and Long-Term Outcome

During follow-up of median 5.3 [4.8–6.1] years, 150 out of 699 (21%) RTR died, of whom 60 (9%) due to a cardiovascular cause. In the same period, 83 out of 699 (12%) RTR experienced graft failure.

**Figure 1:**
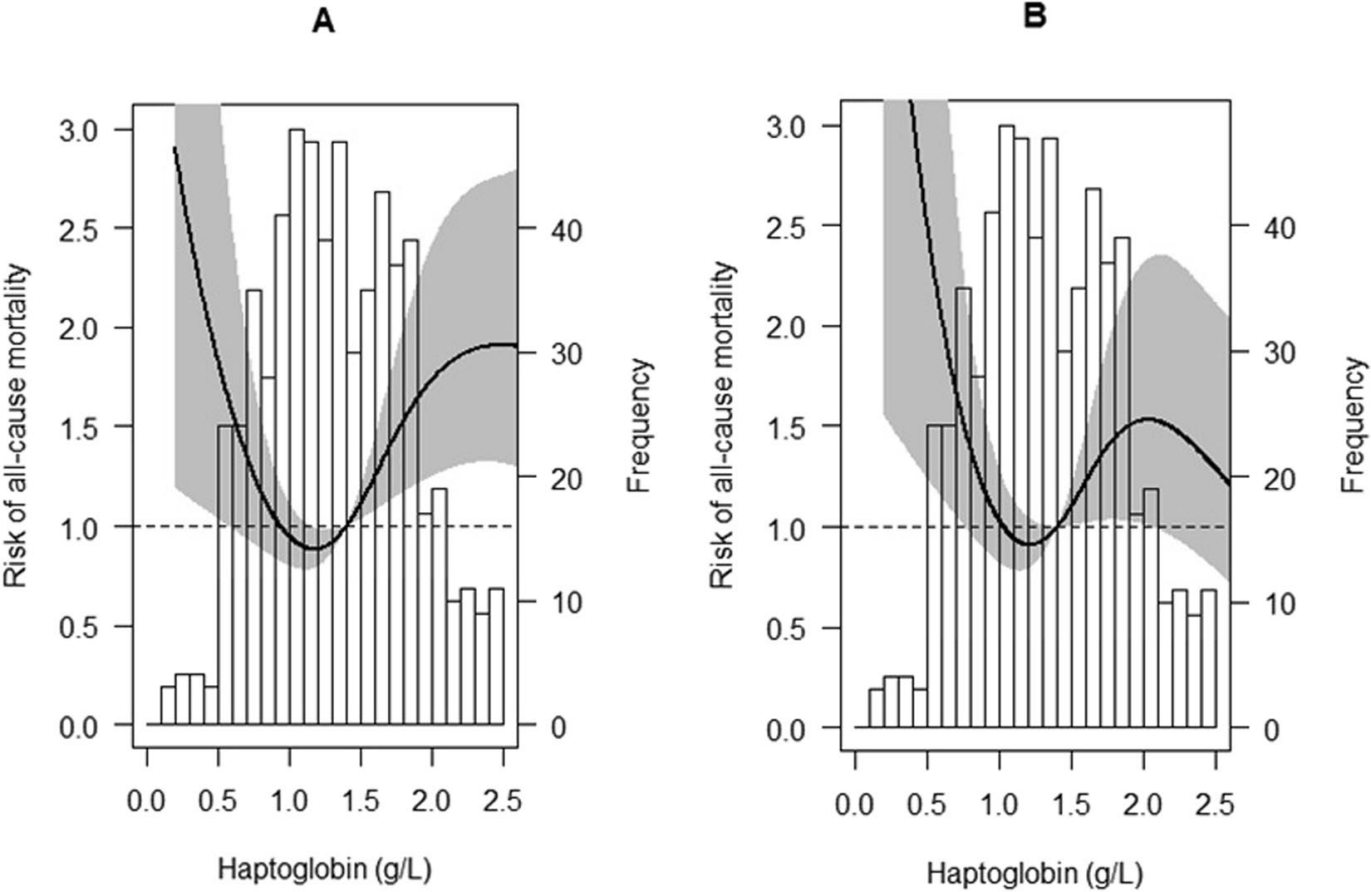
Age- and sex-adjusted (**A**) and fully adjusted (**B**) associations of Hp with all-cause mortality in RTR. The histograms represent frequencies of plasma Hp concentrations, whereas the black lines show the risk (HR) of all-cause mortality. The gray areas represent the appropriate 95% CI’s. The fully adjusted association was adjusted for age, sex, smoking, eGFR, proteinuria, Hp genotype, use of prednisolone, BMI, vitamin B6, waist circumference, triglycerides, HDL-cholesterol, use of statins, SBP, DBP, use of antihypertensive drugs, blood glucose, use of antidiabetic drugs, hs-CRP, procalcitonin, serum albumin, ALAT, ASAT, alkaline phosphatase, gamma glutamyl transferase, plasma MDA, urinary MDA excretion, and serum free thiols.

In age- and sex-adjusted Cox regression models, high (≥2.0 g/L) and low (≤0.9 g/L) circulating Hp concentrations were associated with increased risk of all-cause mortality, Fig. 1A and Table 3. The association of high Hp with mortality, furthermore, was independent of age, sex (model 1), smoking, eGFR, proteinuria (model 2), Hp genotype, use of prednisolone (model 3), and BMI, vitamin B6 (model 4). Adjustment for the components of the MetS, waist circumference, systolic blood pressure (SBP), diastolic blood pressure (DBP), use of antihypertensive drugs, HDL-cholesterol, triglycerides, use of statins, serum glucose, and use of antidiabetic drugs, lowered the hazard ratio (HR) of the fifth quintile by 14% to a significant HR of 1.8 [1.0–3.2] (model 5), as shown in Table 3. Moreover, adjustment for hs-CRP, procalcitonin, and serum albumin further lowered the HR by 29% to a non-significant value of 1.5 [0.8–2.7] (model 6). Adjustment for liver function parameters (model 7), and oxidative stress parameters (model 8) did not materially change the association of high Hp and mortality. In contrast, the association between low circulating Hp concentrations and all-cause mortality, Fig. 1B and Table 3, was independent of the potential confounders.

**Table 3:** Cox regression analyses for the prediction of patient all-cause and cardiovascular mortality based on plasma Hp concentrations. Model 1, adjusted for age, sex; model 2, as model 1, additionally adjusted for smoking, eGFR and proteinuria; model 3, as model 2, additionally adjusted for Hp genotype and use of prednisolone; model 4, as model 2, additionally adjusted for BMI, vitamin B6; model 5, as model 2, additionally adjusted for waist circumference, SBP, DBP, use of antihypertensive drugs, HDL-cholesterol, triglycerides, use of statins, serum glucose, use of antidiabetic drugs; model 6, as model 2, additionally adjusted for hs-CRP, procalcitonin, and serum albumin; model 7, as model 2, additionally adjusted for ALAT, ASAT, alkaline phosphatase, and gamma glutamyl transferase; model 8, as model 2, additionally adjusted for plasma MDA, urinary MDA excretion, and serum free thiols. Hp, haptoglobin; HR, hazard ratio; CI, confidence interval.

	Hp Quintiles (number of events/number of cases)								
	I (27/135)		II (23/149)		III (19/124)	IV (41/163)		V (40/128)	
All-cause mortality	HR [95% CI]	P Value	HR [95% CI]	P Value	Reference	HR [95% CI]	P Value	HR [95% CI]	P Value
Model 1	1.9 [1.0–3.5]	0.03	1.3 [0.7–2.5]	0.34	1.00	1.9 [1.1–3.4]	0.02	2.3 [1.3–4.1]	0.003
Model 2	2.1 [1.2–3.9]	0.01	1.2 [0.6–2.3]	0.57	1.00	1.6 [0.9–2.7]	0.13	2.1 [1.2–3.6]	0.01
Model 3	2.1 [1.2–3.9]	0.02	1.2 [0.7–2.3]	0.50	1.00	1.5 [0.8–2.6]	0.17	1.9 [1.1–3.3]	0.03
Model 4	2.0 [1.1–3.8]	0.02	1.2 [0.6–2.2]	0.66	1.00	1.5 [0.9–2.7]	0.14	2.1 [1.2–3.6]	0.01
Model 5	2.1 [1.1–3.8]	0.02	1.2 [0.7–2.3]	0.53	1.00	1.5 [0.9–2.7]	0.15	1.8 [1.0–3.2]	0.04
Model 6	2.2 [1.2–4.0]	0.02	1.2 [0.7–2.2]	0.59	1.00	1.4 [0.8–2.4]	0.27	1.5 [0.8–2.7]	0.18
Model 7	2.2 [1.2–3.9]	0.01	1.2 [0.6–2.3]	0.55	1.00	1.5 [0.8–2.6]	0.18	1.9 [1.1–3.3]	0.03
Model 8	2.2 [1.2–4.0]	0.01	1.2 [0.7–2.3]	0.52	1.00	1.6 [0.9–2.8]	0.11	2.1 [1.2–3.6]	0.01
Cardiovascular mortality	I (11/135)		II (5/149)		III (6/124)	IV (19/163)		V (19/128)	
Model 1	2.3 [0.9–6.3]	0.10	0.9 [0.3–2.9]	0.83	1.00	2.5 [1.0–6.3]	0.06	3.2 [1.3–8.1]	0.01
Model 2	2.7 [1.0–7.3]	0.06	0.8 [0.2–2.6]	0.68	1.00	1.9 [0.7–4.8]	0.20	2.8 [1.1–7.0]	0.03
Model 3	2.8 [1.0–7.8]	0.04	0.8 [0.2–2.7]	0.72	1.00	1.8 [0.7–4.5]	0.24	2.5 [1.0–6.4]	0.05
Model 4	2.7 [0.9–7.5]	0.07	0.7 [0.2–2.5]	0.62	1.00	2.0 [0.8–5.1]	0.16	2.9 [1.2–7.4]	0.02
Model 5	2.8 [1.0–7.8]	0.05	0.8 [0.2–2.7]	0.72	1.00	1.8 [0.7–4.7]	0.23	2.5 [1.0–6.4]	0.05
Model 6	2.9 [1.0–8.2]	0.04	0.8 [0.2–2.7]	0.75	1.00	1.8 [0.6–4.3]	0.30	1.9 [0.7–4.9]	0.22
Model 7	2.8 [1.0–7.6]	0.05	0.7 [0.2–2.5]	0.63	1.00	1.7 [0.7–4.4]	0.26	2.4 [0.9–6.1]	0.07
Model 8	2.8 [1.0–7.7]	0.05	0.8 [0.2–2.6]	0.72	1.00	2.0 [0.8–5.1]	0.15	2.8 [1.1–7.0]	0.03

Analyses with cardiovascular mortality revealed similar, but higher point estimates for both the fifth and the first Hp quintile across Cox regression models, Table 3. Moreover, adjustment for potential confounders affected the associations of the fifth Hp quintile with cardiovascular mortality in a similar fashion as associations with all-cause mortality.

The continuous, crude association of Hp with graft failure did not achieve significance (P = 0.49). Exclusion of subjects with hemolytic plasma, i.e. 7 RTR, did not materially affect the Cox regression results.

## Discussion

In this study, we showed that circulating Hp under non-hemolytic conditions is associated with components of the MetS, independent of hs-CRP, procalcitonin, and serum albumin, in RTR. Furthermore, we identified that high and low circulating Hp concentrations are associated with an increased risk of all-cause and cardiovascular mortality in this patient setting. While the association of high Hp is attenuated when taking into account inflammation, as measured by hs-CRP, procalcitonin, and serum albumin, the association of low Hp with mortality was independent of potential confounders.

Median circulating Hp concentrations in healthy controls and RTR were in line with previous reports^16–18^. Furthermore, the high prevalence of the MetS among RTR in the present study agreed with previously published literature, which showed that the MetS is present in up to 63% of stable RTR^19–21^.

The exact involvement of Hp as marker of inflammation and adiposity in the MetS is currently unclear^11^. Studies have shown that production of Hp by adipocytes from white adipose tissue is induced by the pro-inflammatory factors interleukin-6 and tumor necrosis factor α^11^. This observation could explain why in the present study adjustment for hs-CRP, procalcitonin, and serum albumin accounted for a relevant part of the association between Hp, waist circumference, and the MetS overall. Notwithstanding, the associations between Hp, waist circumference, and the MetS remained significant after taking into account inflammation. To our knowledge, one non-inflammation related factor has been shown to control Hp gene transcription in adipocytes. It has been demonstrated that dexamethasone is able to increase Hp mRNA levels in cultured adipocytes by approximately threefold, compared to control treatment^22^. While data from this *in vitro* study were limited to dexamethasone, it is conceivable that other corticosteroids have similar effects on Hp production by adipocytes and that this effect might be observed *in vivo*. In line with this reasoning, our data revealed a positive association between use of prednisolone and circulating Hp. However, adjustment for use of prednisolone did not materially affect the associations between Hp and components of the MetS, including waist circumference.

Prospectively, we identified an association between high circulating Hp (≥2.0 g/L) and mortality, in particular cardiovascular mortality. Indeed, high plasma Hp has previously been associated with development of coronary artery disease, possibly through atherosclerotic progression^23^. In the present study, both the associations of circulating Hp with all-cause and with cardiovascular mortality were weakened after adjustment for MetS, and lost significance after adjustment for inflammation. Our data thus suggest that in this patient setting Hp is, prospectively, a marker of inflammation rather than a marker of the MetS.

Interestingly, the associations between low circulating Hp concentrations and mortality were independent of the potential confounders. The causes for these prospective association are unknown, as we have accounted for most factors that have been associated with low circulating Hp concentrations by virtue of the stable nature of our RTR cohort and by controlling for the concerning variables in the statistical analyses. For example, hemolysis, which rapidly decreases circulating Hp concentrations^24^, is virtually absent in our stable RTR cohort and could thus not explain the higher mortality risk for subjects with lower circulating Hp. Other circumstances that are associated with decreased circulating Hp, include Hp genotype^7^ and bone marrow failure^25^. However, adjustment for Hp genotype in the prospective analyses had no material effect. Furthermore, if low Hp is a consequence of bone marrow failure, then a positive relationship between circulating Hp and Hb would be anticipated. Instead, we observed an inverse relationship between Hp and Hb, which argues against ineffective erythropoiesis due to bone marrow failure. Alternatively, low circulating concentration of Hp might be the consequence of malnutrition or liver dysfunction^16,26^. Of the parameters that indicate nutritional status, Hp was associated with BMI and vitamin B6. While RTR with low Hp also had lower BMI, and thus might have had a poorer nutritional status, they discordantly had higher plasma vitamin B6 concentrations, which may be indicative of better nutritional status. Similarly conflicting results were found for the liver function parameters, where ASAT and gamma glutamyl transferase were inversely associated, while alkaline phosphatase was positively associated and alanine aminotransferase (ALAT) was not associated with circulating Hp. Interestingly, adjustment for BMI, vitamin B6, and liver function parameters did not noticeably affect the association of low Hp with mortality. Irrespective of the cause, low circulating Hp concentrations could result in impaired scavenging of the redox active free Hb, which could consequently amplify Hb-mediated oxidative damage to the vasculature^27^. However, RTR with low circulating Hp concentrations (≤0.9 g/L) had similar values of oxidative stress markers as compared to RTR with Hp > 0.9 g/L and adjustment for these markers did not materially affect the association between low circulating Hp and mortality, thus discrediting the hypothesis that RTR with low circulating Hp concentrations experienced increased oxidative stress and that this might have played a role in the prospective associations. Together, the results of these analyses suggest that the association of low Hp with mortality may reflect a constitutional state predisposing to increased susceptibility to cardiovascular disease.

Strengths of this study include the large cohort size of this specific population consisting of well-characterized, stable RTR, in which no cases were lost to follow-up. Extensive information on serum and urinary parameters allowed adjustment for many potential confounders. In addition, the novel ELISA assay used to determine Hp genotype is validated and more sensitive than conventional methods. However, we also acknowledge that this study has limitations. First, the observational study design precludes conclusions on causality. Second, we did not have data on components of the MetS in healthy controls. Therefore, we were unable to assess factors that could explain the potential difference in prevalence of the MetS between healthy controls and RTR. Third, this is a single center study of almost only Caucasian subjects. As a consequence, our results may not be representative for other populations. However, a study involving stable heart transplant recipients reported similar circulating Hp concentrations, which conceivably indicates that increased Hp might be similarly relevant in other stable transplant populations^28^. Future research should address this issue in other transplant populations late after transplantation.

To conclude, Hp is associated with the MetS, independent of inflammation, in RTR. Importantly, both low and high Hp are associated with an increased risk of mortality, predominantly cardiovascular. The association of high Hp with mortality was independent of components of the MetS, but was in part explained by inflammation. This suggests that, prospectively, high Hp can be considered a valid marker of inflammation in RTR. Furthermore, the prospective associations of low Hp were independent of potential confounders, suggesting that constitutionally low Hp is an independent risk factor for mortality in RTR. Further research is warranted to investigate the validity of Hp as marker of the MetS in other populations, including non-renal transplant recipients and the general population, and to identify the mechanisms that underlie the association of low circulating Hp concentrations with long-term mortality in RTR.

## Subjects and Methods

### Study Population

This prospective cohort study was based on a previously described, well-characterized set of 707 RTR^29,30^. Briefly, this cohort included RTR (aged ≥ 18 years) who visited the outpatient clinic of the University Medical Center Groningen (UMCG), Groningen, the Netherlands, between November 2008 and June 2011 and who had a graft that had been functioning for at least 1 year with no history of alcohol and/or drug addiction. Of 707 RTR that provided written informed consent, we excluded subjects with missing biomaterial (i.e. 8 cases) from the statistical analyses, which resulted in 699 cases eligible for analyses. As control group reflecting the general population, we included 149 healthy kidney donors. The study protocol was approved by the UMCG institutional review board (METc 2008/186) and adhered to the Declarations of Helsinki and Istanbul.

### Measurements

Participants were asked to collect a 24-hour urine sample on the day prior to visiting the outpatient clinic. Urine was collected under oil, and chlorhexidine was added as an antiseptic agent. Urinary albumin was quantified using nephelometry (Dade Behring Diagnostic, Marburg, Germany) and total urinary protein concentration was determined by means of the Biuret reaction (MEGA AU 510; Merck Diagnostica, Darmstadt, Germany). Proteinuria was defined as urinary protein excretion ≥0.5 g/24 h.

Upon completion of the 24-hour urine collection, blood was drawn the following morning, and venous blood analyses were assessed immediately thereafter by validated assays. Hp was measured using an automated turbidimetric immunoassay (Roche Diagnostics, Basel, Switzerland). Vitamin B6 was measured as plasma pyridoxal 5′-phosphate by means of a high-performance liquid chromatography (HPLC) assay^31^. Malondialdehyde (MDA) was also quantified by means of an HPLC method and free thiol groups (R-SH) were detected as described previously^32,33^. Free thiol groups were corrected for total serum protein (R-SH/total protein), since serum proteins largely determine the amounts of measurable free thiol groups. Other laboratory measurements, including blood counts, albumin, hs-CRP, lipids, and glucose homeostasis parameters, were performed by automated spectrophotometric routine laboratory methods. Free Hb was quantified in plasma as hemolytic index by a semi-quantitative routine laboratory method with a limit of quantification of 3 µmol/L (Roche Diagnostics, Basel, Switzerland). Hemolysis was identified in samples with hemolytic index > 20. Serum creatinine was determined by means of a modified version of the Jaffé method (MEGA AU 510; Merck Diagnostica). GFR was estimated by applying the Chronic Kidney Disease Epidemiology Collaboration equation^34^. We defined the MetS according to National Cholesterol Education Program Expert Panel on Detection, Evaluation and Treatment of High Blood Cholesterol in Adults (ATP III) criteria^35,36^. In accordance with these criteria, a person has MetS when at least three of the following cardiovascular risk factors are present or medication to control these traits is taken: waist circumference is ≥89 cm for women and ≥102 cm for men, triglyceride level ≥1.7 mmol/L, HDL-cholesterol level ≤1.04 mmol/L for men and ≤1.30 mmol/L in women, SBP ≥130 or DBP ≥85 mm Hg, and fasting blood glucose ≥5.6 mmol/L. Diabetes mellitus was diagnosed according to American Diabetes Association criteria as fasting plasma glucose concentration of at least 7.0 mmol/L or use of antidiabetic medication^37^. Finally, Hp genotype distributions were determined with a novel ELISA method, which was previously described and validated by Levy *et al*.^38^.

### Statistical analyses

Data analyses were performed using SPSS 22.0 for Windows (SPSS Inc., Chicago, IL) and GraphPad Prism version 5.01 for Windows (GraphPad Software, San Diego, CA). Data are presented as mean ± SD for normally distributed data, as median [interquartile range (IQR)] for non-normally distributed data, and as number (percentage) for nominal data. A two-sided P < 0.05 was considered to indicate statistical significance.

Linear regression analyses were employed to investigate differences in Hp between healthy controls and RTR and to assess cross-sectional associations of log-transformed Hp with baseline variables (P_trend_). Multivariable linear regression models were constructed to account for potential confounders, including age, sex, smoking, Hp genotype, eGFR, proteinuria, use of prednisolone, and for inflammation, as assessed by hs-CRP, procalcitonin, and serum albumin^39^. Percentage change in standardized beta was calculated as: ((standardized beta after adjustment – standardized beta before adjustment)/standardized beta before adjustment) × 100.

Several subjects had missing values for one or more baseline variables (i.e., age, sex, time since renal transplantation (Rtx), hs-CRP, eGFR, proteinuria [<0.5%], HbA1c [4.0%], and smoking status [6.2%]). Because excluding subjects with missing values could result in biased prospective results, multiple imputation (fully conditional specification [MCMC]) was employed to obtain five imputed datasets for the Cox regression analyses^40,41^. Rubin’s rules were followed to obtain pooled estimates of the regression coefficients and their standard errors across the imputed datasets^42^.

Prospective associations were explored by means of Cox regression analysis. Prospective data are presented as HR [95% confidence interval]. In these analyses, associations were adjusted in a parallel fashion for potential confounders, including age, sex (models 1), smoking, eGFR, proteinuria (models 2), Hp genotype, use of prednisolone (models 3), BMI, vitamin B6 (models 4), the MetS components waist circumference, systolic blood pressure (SBP), diastolic blood pressure (DBP), use of antihypertensive drugs, high-density lipoprotein (HDL)-cholesterol, triglycerides, use of statins, serum glucose, use of antidiabetic drugs (models 6), the inflammation-related markers hs-CRP, procalcitonin, serum albumin (models 6), liver function parameters ALAT, ASAT, alkaline phosphatase, gamma glutamyl transferase (model 7), and plasma MDA, urinary MDA excretion, and serum free thiols (model 8). Cox regression models were built in a stepwise fashion to avoid over-fitting and to keep the number of predictors in proportion to the number of events^43^. Percentage change in HR was calculated as: ((HR after adjustment – HR before adjustment)/(HR before adjustment-1)) × 100^44^. Proportionality of hazards for covariates was investigated by inspecting the Schoenfeld residuals.

### Clinical End Points

The primary endpoints of this study were all-cause and cardiovascular mortality and death-censored transplant failure, defined as return to dialysis therapy or re-transplantation. The continuous surveillance system of the outpatient program ensures up-to-date information on patient status and cause of graft failure. The cause of graft failure was obtained from patient records and was reviewed by a blinded nephrologist. Cause of death was defined as cardiovascular in origin if death was due to cerebrovascular disease, ischemic heart disease, heart failure, or sudden death. Endpoints were recorded until the end of May 2013 and there was no loss due to follow-up for the primary endpoints.

## Electronic supplementary material

Supplementary Dataset 1
