# Author Correction: Circulating Haptoglobin and Metabolic Syndrome in Renal Transplant Recipients

**DOI:** 10.1038/s41598-018-24791-4

**Published:** 2018-04-20

**Authors:** Isidor Minović, Michele F. Eisenga, Ineke J. Riphagen, Else van den Berg, Jenny Kootstra-Ros, Anne-Roos S. Frenay, Harry van Goor, Gerald Rimbach, Tuba Esatbeyoglu, Andy P. Levy, Carlo A. J. M. Gaillard, Johanna M. Geleijnse, Manfred L. Eggersdorfer, Gerjan J. Navis, Ido P. Kema, Stephan J. L. Bakker

**Affiliations:** 1 Department of Internal Medicine, Division of Nephrology, University of Groningen, University Medical Center Groningen, Hanzeplein 1, 9700 RB Groningen, The Netherlands; 2 Top Institute Food and Nutrition, Nieuwe Kanaal 9-A, 6709 PA Wageningen, The Netherlands; 3 Department of Laboratory Medicine, University of Groningen, University Medical Center Groningen, Hanzeplein 1, 9700 RB Groningen, The Netherlands; 4 Department of Pathology, University of Groningen, University Medical Center Groningen, Hanzeplein 1, 9700 RB Groningen, The Netherlands; 5 Institute of Human Nutrition and Food Science, Christian-Albrechts-University of Kiel, Kiel, Germany; 6 Faculty of Medicine, Technion Institute of Technology, Efron Street 1, Haifa, Israel; 7 Division of Human Nutrition, Wageningen University, Droevendaalsesteeg 4, 6708 PB Wageningen, The Netherlands; 8 DSM Nutritional Products, Wurmisweg 576, 4303 Kaiseraugst, Switzerland

Correction to: *Scientific Reports* 10.1038/s41598-017-14302-2, published online 27 October 2017

The original version of this Article contained a typographical error in the spelling of the author Carlo A.J.M. Gaillard, which was incorrectly given as Carlo AJM Gaillard. This has now been corrected in the PDF and HTML versions of the Article.

