# Supplementary material for: Circulating Haptoglobin and Metabolic Syndrome in Renal Transplant Recipients
Source: Sci Rep. 2017 Oct 27;7:14264. doi: 10.1038/s41598-017-14302-2 (PMC5660219; doi:10.1038/s41598-017-14302-2)
Supplement: Supplementary file 1 — Supplementary Dataset 1 [file 41598_2017_14302_MOESM1_ESM.pdf]

Circulating Haptoglobin and Metabolic Syndrome in Renal Transplant Recipients

Isidor Minović[1,2,3*], Michele F Eisenga[1], Ineke J Riphagen[1], Else van den Berg[1], Jenny Kootstra-Ros[3], Anne-Roos S Frenay[4], Harry van Goor[4], Gerald Rimbach[5], Tuba Esatbeyoglu[5], Andy P Levy[6], Carlo AJM Gaillard[1], Johanna M Geleijnse[7], Manfred L Eggersdorfer[8], Gerjan J Navis[1], Ido P Kema[3], Stephan JL Bakker[1,2]

[1]Department of Internal Medicine, Division of Nephrology, University of Groningen, University Medical Center Groningen, Hanzeplein 1, 9700 RB, Groningen, the Netherlands;

[2]Top Institute Food and Nutrition, Nieuwe Kanaal 9-A, 6709 PA, Wageningen, the Netherlands;

[3]Department of Laboratory Medicine, University of Groningen, University Medical Center Groningen, Hanzeplein 1, 9700 RB, Groningen, the Netherlands;

[4]Department of Pathology, University of Groningen, University Medical Center Groningen, Hanzeplein 1, 9700 RB, Groningen, the Netherlands;

[5]Institute of Human Nutrition and Food Science, Christian-Albrechts-University of Kiel, Kiel, Germany;

[6]Faculty of Medicine, Technion Institute of Technology, Efron Street 1, Haifa, Israel;

[7]Division of Human Nutrition, Wageningen University, Droevendaalsesteeg 4, 6708 PB, Wageningen, the Netherlands;

[8]DSM Nutritional Products, Wurmisweg 576, 4303, Kaiseraugst, Switzerland.

Supplementary Table 1. Baseline characteristics of 149 included healthy controls.

	**Healthy controls (n=149)**
**Demographics**	
Age, years	52±10
Male gender, n (%)	159 (54)**
Weight, kg	79±14
BMI, kg/m^2	25.5 [23.7-28.3]
BMI<21 kg/m^2, n (%)	8 (7)

Waist circumference, cm	96 [92-102]
Smoking status, n (%)	
Current	21 (21)***
Past	21 (21)***
Never	59 (58)***
eGFR, mL/min/1,73m^2	93±13***
Proteinuria, n (%)	0 (0)$^{\#}$
Thrombocytes, 10E9/L	257±60***
MCV, fL	89.7±5.1
Total Hb, mmol/L	8.8±0.8***
Parathyroid hormone, pmol/L	3.3 [2.6-4.2]***
Venous pH	7.37±0.04
Venous HCO_3^-, mmol/L	26.1±1.7***
Hp, g/L	1.1 [0.9-1.4]***
**Dietary intake and nutritional status**	
Total protein intake, g/d	88±24
Animal protein intake, g/d	54±17
Plant protein intake, g/d	34±12
Energy intake, kCal/d	2338±773
Vitamin B12, pmol/L	253 [205-333]**
Vitamin B6, nmol/L	40 [29-60]***
Folic acid, nmol/L	21 [16-27]**
Serum albumin, g/L	45.4±2.7***
**Cardiovascular parameters**	
SBP, mmHg	125±15***
DBP, mmHg	76±9***
Heart rate, bpm	66±9*
Use of antihypertensive drug, n (%)	14 (15)***
Hs-CRP, mg/L	1.0 [0.5-2.2]***
Total cholesterol, mmol/L	5.5±1.2***
LDL-cholesterol, mmol/L	3.6 [2.9-4.4]***
Triglycerides, mmol/L	1.1 [0.8-1.7]***
Use of statin, n (%)	3 (3)***

Hemolytic index>20, n (%)	14 (25)***
Hp 1-1 genotype	25 (24)
Hp 1-2 genotype	55 (52)
Hp 2-2 genotype	25 (24)
**Glucose homeostasis parameters**	
Serum glucose, mmol/L	5.3 [5.1-5.6]
HbA_{1c}, n (%)	5.6±0.3***
Diabetes, n (%)	0 (0)#
Use of antidiabetic drug, n (%)	0 (0)#
**Liver function parameters**	
ALAT, U/L	19 [16-27]
ASAT, U/L	23 [20-27]
Alkaline phosphatase, U/L	68 [58-79]
Gamma glutamyl transferase, U/L	19 [15-33]***
**Antioxidant status parameters**	
Plasma MDA, µmol/L	1.7 [1.0-3.4]***
Urinary MDA excretion, µmol/24h	8.5 [6.3-11.2]***

BMI, body mass index; eGFR, estimated glomerular filtration rate; MCV, mean corpuscular volume; Hb, hemoglobin; Hp, haptoglobin; SBP, systolic blood pressure; DBP, diastolic blood pressure; hs-CRP, high-sensitivity C-reactive protein; LDL, low-density lipoprotein; ALAT, alanine aminotransferase; ASAT, aspartate aminotransferase; MDA, malondialdehyde. *P<0.05, **P<0.01, and ***P<0.001 compared to RTR. #Values could not be tested statistically.
